# The association between functional and morphological assessments of endothelial function in patients with rheumatoid arthritis: a cross-sectional study

**DOI:** 10.1186/ar4287

**Published:** 2013-09-06

**Authors:** Aamer Sandoo, James Hodson, Karen M Douglas, Jacqueline P Smith, George D Kitas

**Affiliations:** 1 Department of Rheumatology, Dudley Group of Hospitals NHS Trust, Russells Hall Hospital, Pensnett Road, Dudley, DY1 2HQ, UK; 2 School of Sport and Exercise Sciences, University of Birmingham, Edgbaston, Birmingham, B15 2TT, UK; 3 Arthritis Research UK Epidemiology Unit, University of Manchester, Oxford Road, Manchester, M13 9PT, UK; 4 Wolfson Computer Laboratory, University Hospital Birmingham NHS Foundation Trust, Queen Elizabeth Hospital Birmingham, Mindelsohn Way, Birmingham, B15 2WB

## Abstract

**Introduction:**

Patients with rheumatoid arthritis (RA) are at an increased risk for cardiovascular disease (CVD). One of the earliest manifestations of CVD is endothelial dysfunction (ED), which can lead to functional and morphological vascular abnormalities. Several non-invasive assessments of vascular function and morphology can be utilised to assess vascular health, but little is known about the association between each of these assessments in patients with RA, and they tend to be used interchangeably in the literature. The objective of the present study was to examine associations between measures of vascular function and morphology in patients with RA.

**Methods:**

A total of 201 RA patients (155 females, median (25^th ^to 75^th ^percentile) age: 67 (59 to 73)) underwent assessments of microvascular endothelium-dependent and endothelium-independent function (laser Doppler imaging with iontophoresis of acetylcholine and sodium-nitroprusside respectively), macrovascular endothelium-dependent and endothelium-independent function (flow-mediated dilatation and glyceryl-trinitrate-mediated dilation respectively), and vascular morphology (pulse wave analysis, carotid intima-media thickness (cIMT), and carotid plaque).

**Results:**

Spearman's correlations revealed that from the functional parameters, only macrovascular endothelium-independent function was inversely associated with cIMT (-0.294 (*P *< 0.001)) after applying the Bonferroni correction for multiple comparisons. For carotid plaque, *t *tests showed that macrovascular endothelium-independent function was lower in patients with plaque than without (15.5 ± 8.3 vs. 23.1 ± 9.1%, *P *= 0.002, respectively).

**Conclusions:**

With the exception of macrovascular endothelium-independent function, all other measures of vascular function were not associated with vascular morphology. This suggests that different assessments of vascular function and morphology in patients with RA reflect quite distinct mechanisms and phases of the atherosclerotic process and should not be used interchangeably.

## Introduction

The endothelium is the innermost layer of the vasculature and is responsible for maintaining an atheroprotective environment within the vessel. Damage to the endothelium from injurious stimuli such as oxidative stress and inflammatory mediators results in endothelial dysfunction (ED) [1]. ED occurs in the early stages of atherosclerosis, and reflects a reduction in the anti-atherogenic molecule, nitric oxide (NO) [2]. In a healthy vessel, NO is tonically released by endothelial cells to modulate vasomotion, and counter the effects of pro-atherogenic molecules such as endothelin-1 (ET-1) [1]. However, continued reduction in NO levels allows ET-1 to increase, resulting in vascular inflammation and subsequent plaque development [3,4]. These vascular alterations can lead to a loss in vasodilatory function, arteriosclerosis, and development of advanced atherosclerosis. Several non-invasive assessments of vascular function and morphology can examine different stages of sub-clinical atherosclerosis and provide useful information on an individual's CVD risk status.

During early atherosclerosis, a reduction in NO bioavailability results in reduced vasodilatation along with the promotion of platelet aggregation and leukocyte adhesion to the vascular wall [5,6]. Assessments which stimulate endothelial release of NO can be used to examine vasodilatory function in the peripheral circulation and serve as an index of early vascular damage [1]. Endothelial function should be examined in the microvasculature and the macrovasculature due to heterogeneous responses to stimulation between these vascular beds [7]. At present, the gold standard assessment of peripheral endothelial function in the microvasculature is laser Doppler imaging with Iontophoresis of NO agonists and in the macrovasculature is flow-mediated dilatation (FMD) (endothelium-dependent) and glyceryl-trinitrate-mediated dilatation (GTN) (endothelium-independent) [1]. These assessments correlate well with assessments of coronary endothelial function [8-10]. Studies have shown that changes in endothelial function are usually transient [11] and can be reversed with treatment [12].

If factors that reduce NO bioavailability are not appropriately controlled then endothelial dysfunction can progress to arteriosclerosis (arterial stiffness) [13]. Arterial stiffness typically occurs in medium to large arteries and is due to degeneration of elastin fibres and deposition of collagen into the vascular wall [14]. A reduction in NO bioavailability and loss of smooth muscle tone also lead to arteriosclerosis, which suggests that this stage of atherosclerosis involves both functional and morphological alterations to the vascular wall [15]. Arterial stiffness can be measured using pulse wave analysis (PWA), which measures the pulse pressure wave in the radial artery and then calculates the central aortic pressure waveform [1]. The consequences of arterial stiffening include insufficient myocardial perfusion leading to angina and myocardial infarction, and left ventricular hypertrophy, which results in heart failure [14]. Arterial stiffness also associates with a number of CVD risk factors such as ageing, smoking, dyslipidemia and hypertension [16], and correlates with assessments of the coronary vasculature [17].

Assessment of advanced, but subclinical, atherosclerosis can be carried out by examination of carotid artery intima-media thickness (cIMT) and by determining the presence of carotid plaques [1]. Thickening of the medial layer of the vessel reflects several atherosclerotic processes that stem from a reduction in NO levels and the concomitant elevation in ET-1 levels; this leads to increased production of pro-inflammatory cytokines, leukocyte adhesion, activation of thrombotic factors, proliferation of smooth muscle cells and formation of lipid-rich plaques [18]. High-resolution B mode ultrasonography is typically used to visualise the carotid artery in different anatomical locations and is a good predictor of future cardiac events in patients with early atherosclerosis [19]. Recent evidence also suggests that determining the susceptibility for plaque rupture may be an additional and important factor to consider when examining subclinical atherosclerosis [20].

Rheumatoid arthritis (RA) is a chronic systemic inflammatory musculoskeletal disease [21]. Patients with RA have an elevated risk of cardiovascular disease (CVD) possibly due to similarities in the inflammatory process in RA and atherosclerosis [22]. There is ample evidence of both functional and morphological abnormalities in the vasculature in RA [12]. At present, little is known on whether assessments of vascular function and morphology can be used interchangeably to provide an index of global endothelial function in RA or whether they should only be used to reflect the distinct stages of atherosclerosis. Indeed, associations between functional and morphological assessments are evident in some cross-sectional studies (see Table 1) [23-25], but studies examining the longitudinal effects of anti-inflammatory medication in RA reveal that some aspects of vascular function, but not morphology, may improve after treatment [26-28]. This suggests that vascular function and morphology might be distinctly affected by RA.

**Table 1:** Studies assessing the relationship between functional and morphological measures of endothelial function.

Authors	Participants	Vascular assessment	Associations
**Healthy**			

Juonala *et al*. 2004 [52]	2109 healthy (age: 32 ± 5 years)	FMD, cIMT	FMD inversely associated with cIMT

Yeboah *et al*. 2008 [29]	2338 healthy (age 78 ± 4)	FMD, cIMT	No significant associations

Koivistoinen *et al*. 2012 [30]	1754 healthy (young) (age: 30 - 45) & 336 healthy (older) (age: 46 - 76)	FMD, PWV, cIMT	No significant associations

Lunder *et al*. 2012 [50]	100 healthy males (age: 42 ± 6)	FMD, GTN, PWV, cIMT	FMD inversely associated with PWV

Irace *et al*. 2006 [31]	77 healthy	FMD, GTN, cIMT	No significant associations

Yan *et al*. 2005 [32]	1,578 healthy males (age: 49 ± 10)	FMD, GTN, cIMT	No significant associations

**Coronary artery disease**			

Rohani *et al*. 2005 [39]	58 CAD (age: 67 ± 9)	FMD, cIMT, carotid plaque	No significant associations

Simova *et al*. 2008 [33]	198 CAD (age: 60 ± 10	FMD, cIMT	FMD inversely associated with cIMT

Furumoto *et al*. 2002 [34]	45 CAD (age: 61 ± 11)	FMD, GTN, cIMT	FMD inversely associated with cIMT

Hashimoto *et al*. 1999 [53]	34 CAD males (age: 61 ± 2) & 33 age-matched healthy controls	FMD, GTN, cIMT	FMD inversely associated with cIMT when both populations combined, and when analysis was performed in healthy controls only & GTN inversely associated with cIMT when both populations combined

Enderle *et al*. 1998 [54]	101 CAD (age: 60 ± 8.6) & 21 non-CAD (age: 57 ± 8.2)	FMD, GTN, cIMT	FMD inversely associated with cIMT

**Diabetes**			

Ravikumar *et al*. 2002 [55]	50 diabetics (age: 54 ± 10) & 50 healthy age- & sex-matched controls	FMD, AIx, cIMT	FMD inversely associated with AIx and cIMT when both populations combined, and when analysis was performed in healthy controls only & FMD inversely associated with AIx in diabetics

**Rheumatoid arthritis**			

Arosio *et al*. 2007 [23]	65 RA females (age: 41 - 52)	FMD, PWV	FMD inversely associated with PWV in RA

Kerekes *et al*. 2008 [25]	52 RA (age: 51 ± 12)	FMD, GTN, cIMT	FMD inversely associated with cIMT

Soltesz *et al*. 2009 [24]	101 patients with autoimmune disease (age: 52 ± 13)	FMD, AIx, PWV, cIMT	FMD inversely associated with AIx, PWV and cIMT

Van Doornum *et al*. 2003 [60]	25 RA (age: 46 ± 8)	FMD, GTN, PWA	FMD not associated with PWA

AIx, augmentation index; cIMT, carotid intima-media thickness; CAD, coronary artery disease; FMD, flow-mediated dilatation; GTN, glyceryl-trinitrate mediated dilatation; PWV, pulse wave velocity; RA, rheumatoid arthritis.

In other populations there is conflicting evidence on whether such assessments correlate with each other (see Table 1). In healthy participants, the majority of studies report no association between functional and morphological vascular assessments [29-32], but some studies in patients with coronary artery disease (CAD) and diabetes find associations between vascular function and morphology [33,34]. However, the findings of these studies are difficult to interpret due to a difference in population characteristics (for example age, gender) and technical aspects of measurement (for example automated or manual vascular boundary detection) between studies.

To the best of our knowledge, there are no studies that have specifically examined correlations between microvascular endothelial function and morphological abnormalities in the vasculature, despite research suggesting that inflammatory mediators produced as a result of microvascular injury may contribute to the formation of atherosclerotic lesions in the macrovessels [35]. In addition, several studies report that endothelium-independent function may be more readily affected by CVD risk factors in patients with cardiovascular disease [36,37] and in RA [38], yet there is a dearth of studies examining the relationship between endothelium-independent function and morphological parameters. Moreover, there is only one small study in patients with CAD that has assessed associations between macrovascular endothelial function and presence of plaque, and found no association [39]. The prevalence of plaque is increased in RA [20], and further research examining vascular predictors of plaque is required.

The aim of the present study was to examine whether microvascular and macrovascular endothelial function associate with arteriosclerosis and carotid atherosclerosis in order to understand whether these distinct assessments of atherosclerosis are independent or related to each other in patients with RA.

## Methods

A total of 201 consecutive RA patients were recruited from the rheumatology outpatient clinics of the Dudley Group NHS Foundation Trust, United Kingdom. All patients met the retrospective application of the 1987 revised RA criteria of the American College of Rheumatology [40]. The study received ethics approval from The Black Country Research Ethics Committee. All participants gave their written informed consent according to the Declaration of Helsinki.

### Protocol

Patients reported to a temperature-controlled vascular laboratory (22°C) after a 12-hour overnight fast. All patients underwent a detailed clinical examination and demographic information was collected by questionnaire. The disease activity score in 28 joints (DAS28) [41] was also calculated. Following this, patients underwent several functional and morphological vascular assessments including laser Doppler imaging with Iontophoresis of acetylcholine (ACh) and sodium nitroprusside (SNP)(microvascular endothelial function), FMD and GTN (macrovascular endothelial function), PWA (augmentation index (AIx)), cIMT as well as assessment of carotid plaque (carotid atherosclerosis).

### Microvascular endothelial function

Endothelial function of the microvasculature was assessed non-invasively using LDI (Moor LDI 2 SIM; Moor Instruments Ltd, Axminster, UK) with iontophoresis of 1% ACh (Miochol-E; Novartis, Frimley, UK) and 1% SNP (Nitroprussiat Fides, Rottapharm Madaus, Barcelona, Spain) in 2.5 ml solution containing 0.5% saline by a single observer (AS). The technique was performed according to previously established guidelines [42] and has been described in detail previously [43]. Briefly, after a baseline scan, 10 scans were recorded during iontophoresis of the vasoactive agents using a 30 μA current, followed by two scans during recovery. This technique has an intra-observer co-efficient of variation (CV) for ACh and SNP of 6.5% and 5.9% respectively in our laboratory.

### Macrovascular endothelial function

Assessment of macrovascular endothelium-dependent function was performed using FMD with high-resolution ultrasonography of the brachial artery (Acuson Antares ultrasound system, Siemens PLC, Camberley, UK) according to previously established guidelines [44]. Endothelium-independent responses were examined by administration of a 500 microgram sublingual glyceryl-trinitrate (GTN) tablet (Alpharma, Barnstaple, UK). The intra-observer CV for the study ultrasonographer (AS) was 10.7% for FMD and 11.8% for GTN assessments respectively. For all vascular tests, endothelial function was expressed as the percentage increase in perfusion or diameter from baseline, and all analysis was carried out offline by AS who was blinded to the identity of the patient.

### Pulse wave analysis

Pulse wave analysis (SphygmoCor Px Pulse Wave Analysis, ScanMed Medical Instruments, Moreton in Marsh, UK) was used to determine AIx as previously described [45]. In brief, an applanation tonometer was positioned over the left radial artery to measure the central aortic waveform. The waveform is calibrated against the brachial blood pressure and contains information on the AIx (calculated as the difference between the second and first systolic peaks and is expressed as a percentage of the pulse pressure). The pressure waveforms in the radial artery were recorded for an 11-second period. The software integrated in the analyser displayed an operator index that reflects the quality of the recorded waveform. If the operator index was low (<75), another reading was taken. Three readings with an operator index >75 were used for analysis. For each parameter, the average of the three readings was calculated.

### Carotid atherosclerosis

High-resolution ultrasonography of the carotid artery was performed by an experienced ultrasonographer (AS) according to previously established guidelines [46] using a 10 MHz linear array probe attached to the same high-resolution ultrasound scanner as for the FMD assessment. The cIMT was defined by determining the thickness between the lines of Pignoli; with the first echogenic line representing the lumen-intima interface, and the second line representing the media-adventitia interface [47]. Assessments of cIMT were performed in the far wall, 1 cm proximal to the carotid bulb at sites free of plaque in both the right and left common carotid arteries using the longitudinal scanning plane. Three measurements were taken on each side, and these were averaged to give the mean IMT for the right and left carotid arteries separately. The IMT from both sides were further averaged to give the overall IMT.

The presence of carotid plaque was identified by scanning the right and left common carotid artery, as well as the internal and external carotid arteries. Carotid plaques were defined as a focal structure encroaching into the arterial lumen of at least 0.5 mm or 50% of surrounding IMT value, or an IMT value of >1.5 mm [46]. The relative echogenicity of the plaques were evaluated using the Artery Measurement Software (AMS, Stockholm, Sweden), which is an automated software specifically developed for analysis of gray-scale median (GSM) as previously described [48]. Analysis of cIMT and the presence of plaque were performed by a single observer (AS) who was blinded to the identity of the patient. The intra-observer CV for cIMT was 8.6%.

### Statistical analysis

Statistical analysis was performed using IBM SPSS version 20 (IBM Corp., Armonk, NY, USA). Continuous variables were tested for normality, and expressed as median (25 to 75^th ^percentile values) or mean ± standard deviation, as applicable, with binary variables expressed as percentages. Spearman's correlation coefficients were used to assess the relationships between functional and morphological parameters of endothelial dysfunction. Associations between presence of carotid plaque (dependent variable) and other vascular parameters (independent variables) were then assessed using *t *tests and Mann-Whitney tests, as appropriate. A *P *value of 0.05 was deemed to be indicative of significance, although this was Bonferroni corrected where necessary to account for multiple comparisons. Power calculations indicated that, for the intended sample size, a correlation coefficient of 0.25 could be detected between functional and structural assessments of endothelial function at 80% power, with alpha of 0.006 (equivalent to 0.05 after Bonferroni adjustment for eight comparisons). For the effect of carotid plaque, detectable differences in the functional assessments of endothelial function ranged from 33% to 50%, at 80% power with alpha of 0.006.

## Results

The patient characteristics are presented in Table 2. The majority of patients were female, had long disease duration but moderate disease activity levels. The median percentage increase in perfusion to ACh was 201% (quartiles: 103 to 390), and for SNP was 132% (56 to 218). In the macrovasculature, mean FMD values were 10.6 ± 7.1%, and the GTN values were 21.7 ± 9.0%. Assessment of PWA revealed that the mean AIx was 32.8 ± 9.1%. Assessment of carotid atherosclerosis yielded a mean cIMT score of 0.69 ± 0.14 mm, with 16 patients showing evidence of plaque in the right carotid artery, and 15 patients showing plaque in the left carotid artery.

**Table 2:** General and disease-related characteristics for the rheumatoid arthritis patients.

	Rheumatoid arthritis patients
*General characteristics*	
Age (years)	67 (59-73)
Sex female N (%)	155 (77)
Body mass index	28 (24-32)
*Disease-related characteristics*	
Disease duration (years)	16 (11-25)
Rheumatoid factor positive N (%)*	113 (81)
DAS28	3.1 (2.5-4.0)
C-reactive protein (mg/l)	3 (2.9-8.5)
Erythrocyte sedimentation rate (mmhr)	12 (5-23)

Results are expressed as median (25th to 75th percentile values) or number (percentage). *Data only available for 140 patients. DAS28, disease activity score in 28 joints.

Spearman correlations were used to examine associations between functional and morphological parameters of endothelial function. This analysis revealed only one significant correlation, after adjustment for multiple comparisons, with GTN being inversely associated with cIMT (Table 3). For carotid plaque, it was found that plaques in the right carotid artery were significantly associated with percentage dilation to GTN (Table 4). Where plaque was present in the right carotid artery, the mean GTN was 15.5 ± 8.3, compared to 23.1 ± 9.1 where no plaque was evident (*P *= 0.002).

**Table 3:** Correlations between functional and structural assessments of endothelial function.

	Arterial stiffness	Carotid intima-media thickness
**Microvascular endothelial function**		
ACh (%)	-0.027 (*P *= 0.729)	-0.159 *(P *= 0.043)*
SNP (%)	0.049 (*P *= 0.524)	-0.055 (*P *= 0.487)
**Macrovascular endothelial function**		
FMD (%)	0.170 (*P *= 0.028)*	-0.131 (*P *= 0.104)
GTN (%)	0.025 (*P *= 0.671)	-0.294 (*P *< 0.001)**

Data displayed as Spearman's rho coefficient (*P *value). *Significant at *P *< 0.05; **significant after Bonferroni adjustment for eight comparisons at *P *< 0.006. ACh, acetylcholine; SNP, sodium nitroprusside; FMD, flow-mediated dilatation; GTN, glyceryl-trinitrate mediated dilatation.

**Table 4:** Effect of plaque on functional assessments of endothelial function.

	Evidence of left plaque			Evidence of right plaque		
	No	Yes	*P *value	No	Yes	*P *value
**Microvascular endothelial function**						
ACh (%)	242.5 (128.3-454.5)	179.5 (95.5-274.5)	0.092	262.0 (130.7-462.0)	152.0 (102.3-214.3)	0.032*
SNP (%)	144.0 (75.5-243.5)	120.0 (23.0-205.0)	0.221	140.5 (74.0-244.0)	143.0 (58.8-223.8)	0.700
**Macrovascular endothelial function**						
FMD (%)	10.8 ± 7.2	10.5 ± 9.1	0.866	10.9 ± 7.4	9.7 ± 6.8	0.537
GTN (%)	22.7 ± 9.2	17.2 ± 9.7	0.061	23.1 ± 9.1	15.5 ± 8.3	0.002**

Microvascular endothelial function data displayed as: median (quartiles), with *P *values from Mann-Whitney tests. Macrovascular endothelial function data displayed as mean ± standard deviation, with *P *values from *t *tests. *Significant at *P *< 0.05; **significant after Bonferroni adjustment for eight comparisons at *P *< 0.006. ACh, acetylcholine; SNP, sodium nitroprusside; FMD, flow-mediated dilatation; GTN, glyceryl-trinitrate mediated dilatation.

## Discussion

The findings of the present study reveal that, in RA, only macrovascular endothelium-independent function associated with carotid atherosclerosis - a marker of advanced morphological vascular alterations. To the best of our knowledge, only two studies have directly examined associations between endothelial function and carotid atherosclerosis in RA, with both studies reporting associations between macrovascular endothelium-dependent function and cIMT [24,25]. However, in one study [24] a heterogeneous sample of patients presenting with a variety of autoimmune diseases (RA: *n *= 14) were recruited, which does not allow for direct comparisons to the present work. In the other study [25], only a small sample of RA patients were assessed (*n *= 52) and macrovascular endothelium-dependent function was considerably lower than those reported in our study (5% and 11% respectively); thus, it is possible that in patients with preserved macrovascular endothelium-dependent function, an association with carotid atherosclerosis is unlikely.

Studies examining the longitudinal effects of anti-inflammatory medication in RA have revealed that microvascular and macrovascular endothelium-dependent function, but not vascular morphology, improved following treatment [26-28]. Furthermore, patients with early RA, showed adverse changes in cIMT at 18-month follow-up relative to baseline, but no change in macrovascular endothelium-dependent function [49]. A similar effect is present when examining functional aspects of the microvasculature and the macrovasculature [38]. Taken together, these findings support the present findings and suggest that RA-associated systemic inflammation may exert differential effects on vascular function and morphology in RA.

Studies in other populations have yielded mixed findings; in healthy participants, five out of the six studies included in Table 1 reported no significant associations between macrovascular endothelium-dependent function and carotid atherosclerosis [29,30,32,50,51]. Only Juonala and colleagues [52] reported an association between macrovascular endothelium-dependent function and carotid atherosclerosis, but their study was confounded by a high CV (26%) for the macrovascular endothelium-dependent function measurement, which may affect the accuracy of their data. In patients with CAD, one study found no association between macrovascular endothelium-dependent function and carotid atherosclerosis [39], with other studies reporting associations between vascular function and morphology [33,34,53,54]. However, these studies do have caveats and should therefore be interpreted with caution. For example, Simova and colleagues [33] found only a weak correlation between functional and morphological parameters and this was no longer significant when adjusting for presence of CAD, number of diseased coronary arteries and percentage coronary artery stenosis [33]. In addition, some of the studies included only small sample sizes (*n *= 34 to 101) [34,53,54], or only found associations when combining patients and healthy controls, but not in patients alone [53,55]. The use of manual detection (rather than automated detection) of the vascular wall also decreased accuracy of obtained data in three of the studies [33,34,53]. Finally, the inconsistent findings between studies may also be due to publication bias in the literature; with studies that find no association between functional and morphological parameters less likely to be published. This may have important implications for future study designs and hypotheses.

The absence of an association between functional and morphological assessments of the vasculature may be due to a time lag between endothelial dysfunction and subsequent lesion development. In the study by Hashimoto and colleagues [53], several participants with atherosclerosis had normal cIMT, but decreased macrovascular endothelium-dependent function, thus highlighting that functional abnormalities may precede morphological abnormalities. Indeed, when examining only functional parameters, microvascular dysfunction often precedes macrovascular dysfunction in RA [56] and in diabetes [57], with both vascular beds being independent from each other in RA [58]. Interestingly, one study reported an association between intima-media thickness of the brachial and carotid arteries, but no association between brachial artery endothelium-dependent function and carotid atherosclerosis [39]. This suggests that functional and morphological abnormalities reflect distinct atherosclerotic processes.

In the present study, macrovascular endothelium-independent function associated with carotid atherosclerosis. Such an association was not surprising as both assessments (to varying degrees) reflect a reduction in smooth muscle relaxation, smooth muscle hypertrophy as well as proliferation of smooth muscle cells [1]. Nevertheless, the association highlights the importance of assessing smooth muscle dysfunction in patients at risk of CVD. In healthy individuals with presence of CVD risk factors, macrovascular endothelium-independent dysfunction can occur independently of endothelium-dependent dysfunction [36]. Furthermore, macrovascular endothelium-independent function, but not macrovascular endothelium-dependent function was related to a reduction in systolic blood pressure (SBP) after 12 and 24 weeks of anti-hypertensive treatment in patients with hypertension [37]. Previous work in RA has also revealed that macrovascular endothelium-independent function associates with several classical CVD risk factors [38], all of which are implicated in the development of carotid artery lesions [59]. Collectively, these findings suggest that abnormalities in macrovascular endothelium-independent function can occur without concomitant abnormalities in macrovascular endothelium-dependent function, and that macrovascular endothelium-independent function is related to morphological changes in the vasculature. However, further detailed prospective studies examining this relationship are warranted.

The strengths of the present work are the inclusion of a large sample of patients, which provided adequate statistical power for the analysis. Additionally, measurements of microvascular and macrovascular endothelium-dependent and -independent function, along with assessments of arteriosclerosis and carotid atherosclerosis allowed detailed characterisation of each stage of atherosclerosis. However, some limitations include the cross-sectional study design, which does not determine cause-effect relationships. Further prospective studies are required to see which measures of endothelial function and vascular morphology are able to predict the development of future cardiac events.

In conclusion, the present work highlights that, with the exception of macrovascular endothelium-independent function, assessments of vascular function and morphology cannot be used interchangeably to assess vascular health. Examination of endothelial function should incorporate functional and morphological assessments to provide information on global endothelial dysfunction.

## Abbreviations

ACh: acetylcholine; AIx: augmentation index; CAD: coronary artery disease; cIMT: carotid intima-media thickness; CVD: cardiovascular disease; CV: co-efficient of variation; ED: endothelial dysfunction; ET-1: endothelin-1; FMD: flow-mediated dilatation; GTN: glyceryl-trinitrate-mediated dilatation; NO: nitric oxide; PWA: pulse wave analysis; PWV: pulse wave velocity; RA: rheumatoid arthritis: SNP: sodium nitroprusside.

## Competing interests

The authors declare that they have no competing interests.

## Authors' contributions

AS participated in the design of the study, recruited patients, performed the vascular assessments, conducted data analysis and drafted the manuscript. JH performed the statistical analysis. KD participated in the design of the study. JPS performed laboratory analysis of blood samples. GK participated in the design of the study and helped with drafting the manuscript. All authors have read and approved the final manuscript.
